# Systemic inflammatory response in the pediatric emergency department: a common phenomenon that does not predict severe illness

**DOI:** 10.1186/cc14041

**Published:** 2014-12-03

**Authors:** CPZ Foo, G Sangha, J Seabrook, J Foster

**Affiliations:** 1 Department of Paediatrics, Western University, London, Canada; 2 Children's Health Research Institute, London Health Sciences Centre, London, Canada; 3 Division of Food and Nutritional Sciences, Brescia University College, London, Canada

## Introduction

Systemic inflammatory response syndrome (SIRS) was defined in pediatrics primarily to aid in research subject identification. There is a paucity of data on the clinical utility of SIRS in the pediatric population. We aimed to determine the incidence of SIRS in children presenting to the pediatric emergency department (PED) of a mid-sized Canadian tertiary care center and, secondarily, to examine the sensitivity and specificity of SIRS for predicting infection, the range of clinical entities presenting with SIRS, and outcomes of children with SIRS.

## Methods

We conducted a prospective cohort study of all children from birth to 18 years presenting to the PED on 16 days distributed evenly over 1 year. Charts of all patients presenting to the PED on study days were reviewed to determine the presence of SIRS and sepsis. Three pediatricians adjudicated all cases of presumed infection. All patients were followed for 1 week to determine outcomes.

## Results

The incidence of SIRS was 14.3% (*n *= 202 of 1,416). The rate of documented or presumed infection in the entire population was 37.1% (*n *= 525), and in SIRS patients was 81.2% (*n *= 164). Therefore, 11.6% of the population (*n *= 164) met criteria for sepsis, one patient had severe sepsis, and there were no cases of septic shock. Sensitivity and specificity of SIRS for predicting infection was 31.2% (95% CI: 27.3 to 35.4%) and 95.7% (95% CI: 94.2 to 97.0%) respectively. No difference in sensitivity or specificity was seen when cases were separated by age. Patients with SIRS had a higher risk of requiring hospital admission with a relative risk of 2.8 (95% CI: 2.0 to 4.1, *P *< 0.0001). Although children with SIRS stayed in hospital statistically longer (*P *< 0.001), the median length of stay for both groups was ≤1 day. However, 28.7% (*n *= 47) of patients meeting definition of sepsis had nonsevere infection types (for example, acute otitis media, viral upper respiratory tract infection); 72.3% of patients with SIRS and 75% of patients meeting sepsis criteria were discharged home from the PED without return visits.

## Conclusion

While SIRS has a high specificity for infection, its poor sensitivity suggests a risk of missing infection if used as a screening tool. Although patients with SIRS have an increased risk of admission, the current definition of sepsis may result in over-inclusion of patients with nonsevere disease.

